# Structural and Physical Properties of Chitosan Films Containing UV-Driven *In Situ* Growth of Silver Nanoparticles

**DOI:** 10.1021/acsomega.5c09873

**Published:** 2026-03-30

**Authors:** Daniele Costa, Mariafrancesca Cascione, Valeria De Matteis, Riccardo Di Corato, Nunzia Gallo, Stefania Villani, Christian Demitri, Pietro Alifano, Alessandro Sannino, Gianaurelio Cuniberti, Rosaria Rinaldi

**Affiliations:** † Department of Mathematics and Physics “Ennio De Giorgi”, 18976 University of Salento, Via Arnesano, Lecce 73100, Italy; ‡ 312432 Institute for Microelectronics and Microsystems (IMM) CNR, Via Monteroni, Lecce 73100, Italy; § Department of Experimental Medicine, 18976 University of Salento, Via Barsanti, Arnesano Lecce 73100, Italy; ∥ Center of Biomolecular Nanotechnologies, Istituto Italiano di Tecnologia, Via Monteroni, Lecce 73100, Italy; ⊥ Department of Engineering for Innovation, 18976 University of Salento, Via Monteroni, Lecce 73100, Italy; # Institute for Materials Science and Max Bergmann Center for Biomaterials, 9169 TUD Dresden University of Technology, Dresden 01069, Germany

## Abstract

Chitosan (CS), a naturally abundant biopolymer mainly sourced from marine crustacean waste, has emerged as a sustainable alternative to conventional, nonbiodegradable synthetic polymers in food packaging due to its intrinsic biocompatibility, nontoxicity, higher sustainability, biodegradability, and excellent film-forming ability. Furthermore, CS exhibits a remarkable dual role as both a reducing and capping agent in the green synthesis of silver nanoparticles (AgNPs). In the present work, CS-based nanocomposite films embedded with green AgNPs were produced via a straightforward one-step UV photoreduction method. CS simultaneously acted as a reducing and capping agent for AgNPs obtained *in situ* and as a rigid matrix for their confinement. By varying the UV exposure times (15, 45, and 90 min), the morphology and size of the NPs were characterized using transmission electron microscopy (TEM), revealing a predominantly spherical shape with an average diameter of ∼60 nm. In parallel, each resulting film was thoroughly analyzed using different techniques to evaluate the impact of UV radiation and *in situ* AgNP formation on the polymers’ physical and structural properties, including wettability, moisture content, swelling degree, water solubility, surface morphology, roughness, and mechanical behavior. In addition, antibacterial efficacy was assessed against both *Escherichia coli* and *Staphylococcus aureus*, demonstrating inhibition of both Gram-positive and Gram-negative strains. Moreover, the silver ion release in aqueous media (pH 7) was quantified via ICP-OES. The simplicity, scalability, and effectiveness of the proposed method underscore the potential of the AgNPs@CS film as a sustainable antibacterial material for next-generation food packaging solutions.

## Graphical abstract

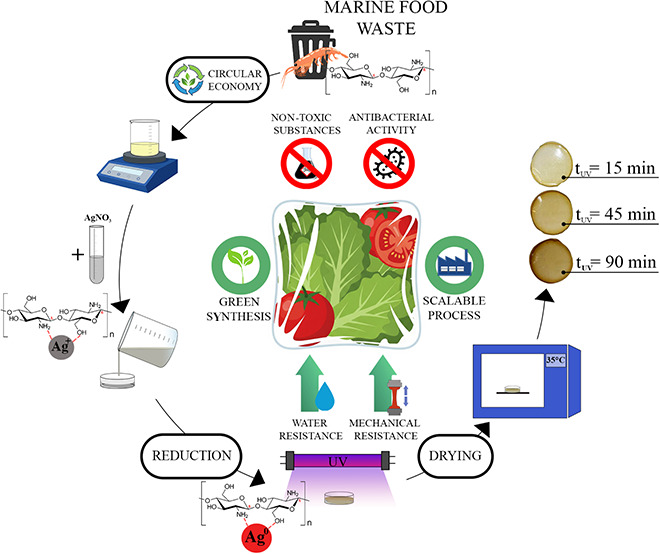

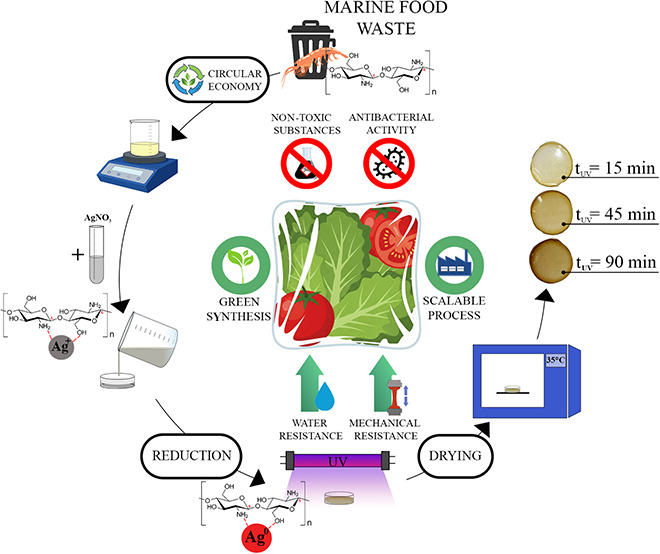

## 1 Introduction

In recent years, biopolymers have gained significant interest as sustainable alternatives to conventional petroleum-based materials, particularly in wound dressing,^1^ flexible electronics and photonics,^2^ water treatment,^3^ biosensing,^4^ and food packaging fields.^5^ Derived from renewable natural sources, including starch, polybutylene, polylactic acid (PLA), cellulose, sodium alginate, pectin, and chitosan (CS), the biopolymers offer the advantages of biodegradability, biocompatibility, and low toxicity.^6–11^ Among them, CS, obtained through the deacetylation of chitin (the second most abundant natural source after cellulose), has emerged as a versatile platform due to its remarkable film-forming ability.^12,13^ Incorporating or embedding organic or inorganic nanomaterials (1–100 nm) into biopolymer matrices can significantly enhance their performance. The high surface area-to-volume ratio of nanofillers strengthens interfacial adhesion, thereby yielding marked increases in tensile modulus, thermal stability (e.g., higher glass transition and decomposition temperatures), and gas/moisture barrier performance.^13–17^ Metallic and metal oxide NPs, particularly AgNPs, are increasingly investigated as functional additives in biopolymer-based nanocomposites. Owing to their engineered physicochemical properties and demonstrated antimicrobial and antioxidant activities, these nanocomposites have emerged as promising candidates for active food packaging applications. The suppression of microbial growth and oxidative degradation has been demonstrated for AgNPs, thereby extending the shelf life of perishable products.^18,19^ A critical challenge, however, lies in identifying scalable fabrication techniques compatible with biopolymers, as these are generally unsuitable for conventional processing methods developed for synthetic polymers.^20^

AgNPs are typically synthesized via the chemical reduction of silver salts in aqueous media, forming a stable colloidal suspension. They can be incorporated into biopolymer matrices using ex-situ methods. Specifically, preformed AgNPs are added to a biopolymer solution and mixed, usually by stirring or sonication, to ensure that the NPs are evenly dispersed throughout the polymer matrix. In this context, the polymer functions as a dispersion medium, and nanocomposite films are subsequently fabricated using the solution casting method.^21–25^ However, this approach generally requires additional reducing and capping agents to synthesize and stabilize the NPs. Moreover, it is often restricted to small-scale applications due to challenges such as NP aggregation, reduced nanofiller efficacy, and suboptimal interfacial bonding between the matrix and the nanophase. These factors may impair the overall material performance.^26,27^ In contrast, *in situ* synthesis techniques provide a one-pot, green processing route, wherein the biopolymer itself simultaneously serves as both a reducing and stabilizing agent for metal ions and NPs’ growth.^28^ This method enables the direct formation of metal–polymer nanocomposites with homogeneously distributed NPs. Furthermore, it eliminates the need for NP isolation, purification, or the addition of potentially hazardous surfactants. The result is a significant reduction in processing time and solvent use, along with the formation of well-dispersed, size-controlled NPs intimately embedded within the polymer network. These characteristics contribute to improved mechanical strength, optical properties, and functional performance of the resulting films, besides their sustainability and cost-effectiveness.^17,29^

CS has been widely employed as a capping and reducing agent in the *in situ* synthesis of AgNPs through several approaches. These include spontaneous reduction at room temperature,^30–32^ thermally induced reduction,^33^ reduction via chemical or biological agents,^22,34,35^ gamma irradiation,^36^ and UV radiation.^37–42^ Among these, UV-induced photoreduction has been extensively investigated as an effective strategy for generating AgNPs within CS matrices. Boufi et al.^42^ synthesized both AgNPs and gold nanoparticles (AuNPs) by irradiating a mixture of metal salt precursors and aqueous CS solution with a UV lamp emitting in the 200–400 nm range. Similarly, Devendrapandi et al.^39^ utilized a natural light source to induce AgNPs in CS solution. In other studies, CS films were first impregnated with silver precursors and subsequently exposed to UV interaction. Examples include the work conducted by Mehrabanian et al.,^40^ who used laser pulses from a Nd:YAG laser (λ = 355 nm), and the work published by Chen et al.,^43^ in which a UV mercury lamp (λ = 254 nm) was used for the same purpose. However, it should be taken into account that CS is subjected to photodegradation when it interacts with radiation at wavelengths λ < 360 nm, while little or no evidence has been reported for wavelengths λ > 360 nm (UVA).^44–46^

To the best of our knowledge, no prior studies have demonstrated the dual function of CS as both a reducing/capping agent and a solid-state film-forming matrix under mild UVA irradiation.

In our work, we investigated a one-step, *in situ* green synthesis route for the fabrication of CS@AgNP nanocomposite films, employing low-power UVA irradiation (λ ≈ 365 nm) to induce NP formation directly within the CS matrix. The resulting nanocomposite films were extensively characterized with respect to their physical properties and antibacterial performance. Furthermore, the individual and combined effects of AgNO_3_ salt and UVA exposure on the structural and functional properties of CS films were systematically evaluated, revealing notable enhancements in mechanical and barrier characteristics, thereby advancing their potential as active materials for food packaging applications. This work offers a new sustainable, easily scalable, and solvent-free approach for the development of multifunctional biopolymer-based nanocomposites, with valuable insights into their structure–property relationship and their applicability in active food packaging technologies.

## 2 Materials and Methods

### 2.1 Materials

CS medium molecular weight (powder, deacetylation degree ≥75%, molecular weight 190–310 kDa, CAS 9012-76-4, Sigma-Aldrich, Dorset, UK); silver nitrate (AgNO_3_, molecular weight 169,87 g/mol, CAS 7761-88-8, Honeywall Fluka, USA); acetic acid (CH_3_CO_2_H, ACS reagent, ≥99.7%, CAS 64-19-7, Sigma-Aldrich, Dorset, UK); nitric acid (HNO_3_, ≥69%, CAS 7697-37-2, Honeywell Fluka, USA); UV lamps (2 × lamps TL 8W BLB 1FM/10 × 25CC, Philips, central wavelength λ = 365 nm); ultrapure water (Milli-Q, Barnstead Smart2Pure water purification system, Thermo Scientific, USA).

### 2.2 Preparation of Chitosan Films

A 2% (w/v) CS solution was prepared by dissolving 200 mg of CS powder in 10 mL of a 1% acetic acid solution under stirring at 350 rpm for 24 h. The obtained viscous solution was centrifuged at 3400 rpm for 30 min to remove any undissolved residues. For the control sample (CTRL), 2 g of the solution was poured into a polystyrene plate (with a diameter of 35 mm) at a casting density of 0.2 g/cm^2^, and placed in a ventilated oven at 35 °C for 48 h. The dried film was peeled off and left in a dark, controlled room at (18 ± 2) °C and a relative humidity of (50 ± 5)% for 1 week. A UV-exposed control sample (CS/UV) was prepared following the same procedure, except for exposure to UV radiation (λ = 365 nm), at a distance of 12.5 cm, for 1.5 h prior to the drying step.

### 2.3 Preparation of Chitosan-Silver Nanocomposite Films

CS@AgNP nanocomposites were prepared by first dissolving 200 mg of CS powder in 9 mL of 1% acetic acid solution under stirring at 350 rpm for 24 h. Subsequently, 1 mL of freshly prepared 10 mM AgNO_3_ solution was added, yielding a final concentration of 2% CS and 1 mM AgNO_3_. The mixture was stirred for an additional 3 h at 500 rpm. The obtained pale mixture was centrifugated at 3400 rpm for 30 min, and the pellet was discarded. From the supernatant, 2 g of solution was poured into 35 mm polystyrene plate, resulting in a casting density of 0.2 g/cm^2^ for each sample. UV irradiation was applied for 15, 45, and 90 min to induce NP formation, resulting in samples CS15, CS45, and CS90, respectively. Then, the CS@AgNP films were dried following the same procedure used for pure CS films. The unexposed sample, serving as a control, was designated as CS0.

### 2.4 Characterization Techniques

#### 2.4.1 Transmission Electron Microscopy (TEM)

The AgNPs’ morphology and distribution were analyzed using a JEOL JEM-1011 transmission electron microscope (TEM, Jeol Ltd., Tokyo, Japan) operating at an accelerating voltage of 100 kV. For this purpose, 2 g of CS@Ag solution was poured into a glass vial, exposed to UV radiation for 15, 45, and 90 min, respectively, and then allowed to rest for 24 h. The solutions were subsequently centrifuged at 13000 rpm for 30 min to collect the NP-containing pellets. Additional centrifugation steps were carried out to eliminate the residual polymer content. The obtained pellets were resuspended in 1 mL of Milli-Q water and deposited onto copper grids for TEM measurements. NP size was estimated by ImageJ v.1.53 (National Institutes of Health, USA) software on ∼60 individual particles.

#### 2.4.2 UV–Vis Spectroscopy

UV–vis spectroscopic analysis was performed on free-standing films using a Lambda 900 UV–vis/NIR spectrometer (PerkinElmer, USA), with a UV/vis wavelength accuracy of ±0.08 nm. Baseline calibration was carried out prior to measurement using an empty sample holder. Spectra were recorded in the range of 248–800 nm with a step of 4 nm. Data were processed and plotted using OriginPro software (OriginLab, version 8.5, Northampton, MA, USA). Samples were analyzed 3 times.

#### 2.4.3 Wettability

Surface wettability of all samples was evaluated by using static contact angle (C.A.) measurements with the sessile drop technique. An optical tensiometer (Attension Biolin Scientific, Finland) equipped with a CMOS 1/2.3 in. USB 3.0 digital camera was used. Ten drops (3 μL each) of Milli-Q water were deposited on each film surface, and images were acquired 0.5 s after deposition to minimize transient effects. Measurements were repeated six times on fresh samples at room temperature (22 ± 2) °C and relative humidity of (60 ± 5)%, for a total of approximately *n* = 60 measurements per sample type.

#### 2.4.4 Moisture Content (MC%), Swelling Degree (SD%), and Solubility (S%)

Moisture content (MC%), swelling degree (SD%), and solubility (S%) were determined following the protocol described by Homez-Jara et al.,^47^ with minor modifications. Briefly, films were prepared as previously described by casting the poured solutions into 16 mm diameter polystyrene dishes at a concentration of 0.2 g/cm^2^.

After drying for at least 10 days at (18 ± 2) °C and (50 ± 5) RH%, the samples were weighed (M_0_) using an analytical balance (Sartorius Competence CPA225D, Sartorius AG, Germany). Subsequently, the samples were dried in an oven at 100 °C for 24 h to remove adsorbed water and weighed again (M_1_). Thereafter, they were immersed in 4 mL of Milli-Q water at room temperature for 24 h. After gently drying with filter paper, the samples were weighed (M_2_), then dried again at 100 °C for 24 h and weighed a final time (M_3_). Each sample type was tested eight times. The values for MC%, SD%, and S% were respectively calculated using the following eqs 1,2 and 3:

1 $$MC\%=\frac{M_{0}-M_{1}}{M_{0}}\times 100$$

2 $$SD\%=\frac{M_{2}-M_{1}}{M_{1}}\times 100$$

3 $$S\%=\frac{M_{1}-M_{3}}{M_{1}}\times 100$$

#### 2.4.5 Atomic Force Microscopy (AFM)

Topographical characterizations were performed using AFM (Bioscope Catalyst, Bruker Inc., Santa Barbara, CA, USA), implemented on an inverted optical microscope (Zeiss Observer Z1, Zeiss, Jena, Germany). Samples were prepared on microscope slides following the previously described method. They were left to dry under controlled temperature and humidity conditions of (18 ± 2) °C and (50 ± 5) RH%, respectively. The AFM acquisition was obtained using a silicon nitride probe (SNL-10 A, Bruker Inc., USA), consisting of the triangular geometry of a silicon tip. Each scan was performed at 0.4 Hz on areas of (50 × 50) μm^2^ with a resolution of 256 lines × 256 dots. NanoScope Analysis software (Bruker Inc., USA) was used to quantify the roughness parameter of roughness, expressed as R_q_ (root-mean-square roughness), on at least 10 regions of (4.5 × 4.5) μm^2^ for *n* = 3 replicates.

#### 2.4.6 Mechanical Tests

The mechanical properties of CS-based films were evaluated through tensile tests in accordance with the ASTM D882 standard practice, using a ZwickLine universal testing machine (Zwick/Roell, Ulm, Germany) equipped with a 100 N load cell. Briefly, CS-based films were cut into specimens of approximately 100 × 10 mm and conditioned at 25 °C and 50% relative humidity for no less than 40 h prior to testing. Uniaxial tensile tests were performed under displacement control until failure, with a preload of 0.01 N and a test speed of 12.5 mm/min. Film thickness was measured with a Dino-Lite digital microscope (AnMo Electronics Corporation, New Taipei City, Taiwan). Each sample type was tested in six replicates.

#### 2.4.7 Antimicrobial Activity of Films by Agar Diffusion Method

*Escherichia coli* FB8 ^48^ and *Staphylococcus aureus* SA01 ^49,50^ were selected as representative Gram-negative and Gram-positive strains, respectively, to assess the antimicrobial properties of the CS-based materials. Both strains were grown in Luria–Bertani (LB) medium, containing 1% NaCl, 1% Tryptone, and 0.5% Yeast Extract, with the addition of 1.5% Agar for solid medium. The medium was sterilized by autoclaving at 121 °C for 20 min prior to use. Bacterial preinoculum was prepared by suspending a colony isolated from a fresh agar plate (24–72 h) into LB broth, followed by overnight incubation at 37 °C with stirring at 120 rpm. The antibacterial efficacy of the CS-based films was evaluated using the agar diffusion method, following the guidelines outlined in the CLSI M100 – Performance Standards for Antimicrobial Susceptibility Testing (M02 – disk diffusion)^51^ (ISBN 978-1-68440-067-6). The plates were seeded with the bacterial inoculum equivalent to 0.5 McFarland standard (O.D. ∼0.08 measured at 600 nm). Then, each film sample was cut into discs (6 mm diameter), placed on the agar plates, and incubated at 37 °C for 24 hours. At the end of the incubation, the diameter of the inhibition zones was measured using ImageJ v.1.53 software (National Institutes of Health, USA). All experiments were conducted in triplicate. The error *ε* was calculated as

4 $$\varepsilon =\frac{X_{\max}-X_{\min}}{2}$$

where *X*_max_ and *X*_min_ are, respectively, the highest and the lowest measured values.

#### 2.4.8 Ion Release

The release of silver ions (Ag^+^) from the CS@Ag complex films and CS@AgNP nanocomposites was quantified using Inductively Coupled Plasma Optical Emission Spectroscopy (ICP-OES) (Agilent 720-ES, Agilent Technologies, United States). Circular film samples (16 mm in diameter) were soaked in 1 mL of Milli-Q water (pH = 7) and kept at room temperature for 1, 7, and 21 days. At each time point, the supernatant was collected and acid-digested overnight with 10% HNO_3_ (v/v). Prior to ICP-OES measurement, all samples were further diluted (1:5) with Milli-Q water to ensure accurate quantification within the calibration range.

#### 2.4.9 Statistical Analysis

Unless otherwise specified, all results were reported as Means ± SD. Statistical analyses were performed using OriginPro software (OriginLab, version 8.5, Northampton, MA, USA). Specifically, parametric (ANOVA and *t*-test) and nonparametric (Kruskal–Wallis H and Mann–Whitney) tests were used for group or pairwise comparisons. Differences were considered statistically significant at *p* < 0.05. Statistical test results were reported in the form of graphs alongside each histogram and in the Supplementary (S1) file.

## 3 Results and Discussion

The packaging sector is a major contributor to the proliferation of single-use plastic materials, of which only 9% are effectively recovered and recycled.^52^ The majority of the remainder is dispersed into the environment, posing serious threats to both human and animal health.^6^ As a response, sustainable alternatives based on natural polymers are being explored, often requiring functional improvements through the incorporation of inorganic nanomaterials, which allow precise tailoring of their physicochemical properties.^5^ Among these, metal-based nanomaterials such as AgNPs can be synthesized following chemical and physical routes. However, the most promising and increasingly explored approach involves green synthesis, leveraging natural sources such as algae, plants, bacteria, fungi, or polymers to obtain safer and more sustainable NPs.^22,53,54^ Typically, the subsequent integration of such NPs into a polymeric matrix is achieved via dispersion techniques; however, these methods frequently result in nonuniform distributions and NP agglomeration, ultimately reducing their functional performance.^27^

CS is a biopolymer with strong chelating abilities toward various metal ions, primarily due to the presence of amino and hydroxyl functional groups within its molecular structure. In particular, the amino groups serve as coordination sites for Ag^+^, facilitating the formation of a CS–Ag complex.^55^ It has been previously demonstrated that the reduction of Ag^+^ to Ag^0^ can be activated by UV radiation through the formation of hydrated electrons, free radicals, or charge transfer. However, since hydrated electrons typically require ionizing radiation for formation in aqueous media,^56^ the most plausible mechanism under UVA exposure involves radical generation and/or a process of ligand-to-metal charge transfer.^57,58^

In the present work, a novel one-step and eco-friendly procedure was introduced to fabricate *in situ* CS@AgNP nanocomposite films using UV radiation, without the need for an additional reducing agent. Although the formation of AgNPs in CS solution under UV irradiation has been extensively reported in the literature,^38,39,41,42^ most of these studies have focused solely on NP synthesis in CS media, without addressing the development of nanocomposite films. In other cases, Ag incorporation was achieved by impregnating preformed CS films with a precursor solution followed by UV exposure, aiming to bind silver ions (Ag^+^) to the polymerized CS matrix.^40,43^ However, the immersion of CS films in aqueous solutions can, in some instances, lead to their dissolution or structural degradation, compromising their applicability.^44,59,60^

To date, no prior study has simultaneously employed CS as both a reducing agent under UV irradiation and a structural matrix for the *in situ* growth of AgNPs to manufacture thin solid films. A similar work was conducted by Bousalem et al.,^37^ where, however, the AgNPs were *in situ* reduced in an alginate solution and consequently additivated in chitosan for a procedure that recalls the *ex situ* ones

In our approach, CS powder of medium molecular weight was dissolved in a 1% acetic acid solution without the addition of chemical agents for reducing Ag^+^ into Ag^0^ species. The resulting viscous yellowish CS solution (Figure 1a) was used to prepare two different control samples: CTRL and CS/UV. The latter consisted of a solution poured into a 35 mm polystyrene dish and exposed to UV radiation at 365 nm for 90 min. This sample serves to distinguish between the effects of UV exposure alone and those induced by AgNP formation. The 90-min time was selected to match the longest UV exposure used in the subsequent nanocomposites’ preparation.

**Figure 1:**
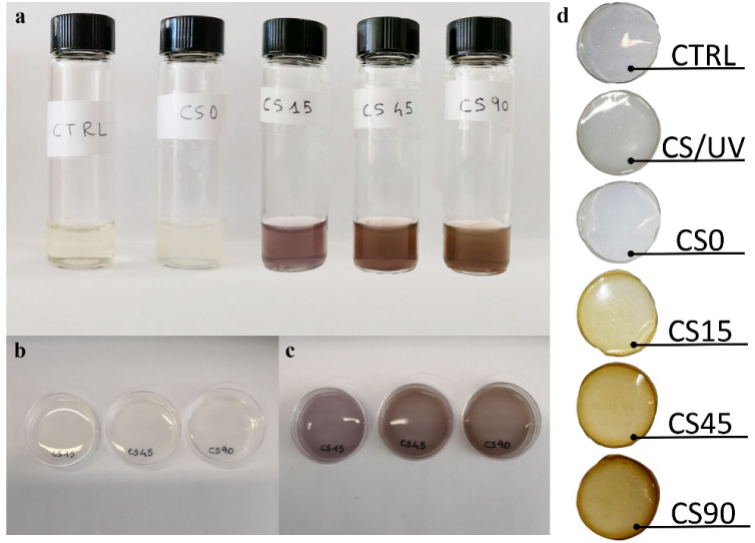
(a) Vials containing 2 g of CS solution after each step of the process. From left to right: CS solution used in CTRL and CS/UV samples, the CS@AgNO_3_ solution used in CS0 samples, the solution CS@AgNO_3_ after 15, 45, and 90 min of exposure to UV radiation, respectively CS15, CS45, and CS90; (b) CS@AgNO_3_ solution in polystyrene dishes before UV exposure; (c) CS@AgNO_3_ UV-irradiated for 15, 45, and 90 min within polystyrene dishes, respectively CS15, CS45, and CS90; (d) polymerized and dried films after 1 week.

Upon the addition of AgNO_3_ to the CS liquid solution, and after 3 h of stirring in the dark, the resulting complex appeared optically pale white (Figure 1 a,b). To prevent unintentional photoreduction, the CS/AgNO_3_ solutions were transferred into polystyrene dishes and stored in an oven at 35 °C in the absence of light to obtain the CS0 samples. No color changes were observed prior to UV exposure, confirming that the reduction of Ag^+^ ions was strictly UV-exposure dependent. Following irradiation, the solution turned reddish-brown, suggesting AgNP formation (Figure 1c). To evaluate the extent of ion reduction, three different UV exposure times—15, 45, and 90 min—were applied, yielding CS15, CS45, and CS90 samples, respectively.

A part of the previously prepared CS@AgNO_3_ solution was exposed to UV radiation and subjected to several centrifugation steps, as reported in section 2.4.1, in order to verify the presence of NPs. The polymer-deprived pellets were dispersed in Milli-Q water and then deposited onto copper grids for the subsequent TEM analysis. In all samples, the NPs appeared almost spherical and were surrounded by a low-contrast halo, which can be attributed to the presence of the CS polymer (Figure 2 top). The size analysis conducted on approximately *n* = 60 individual particles showed an average size inversely proportional to the UV irradiation time. Indeed, the NPs obtained after UV irradiation for 15 min were around (70 ± 16) nm (Figure 2a), in contrast to those obtained after 45 and 90 min, which were, respectively, (55 ± 22) nm and (57 ± 9) nm, as reported in Figure 2b and c (*p* < 0.05) (see S1). In addition, the appearance of a NPs’ population with a reduced size of (29 ± 10) nm (*p* < 0.05) (Figure 2c,f) after UV exposure for 90 min can be attributed to the photofragmentation of AgNPs.^61,62^

**Figure 2:**
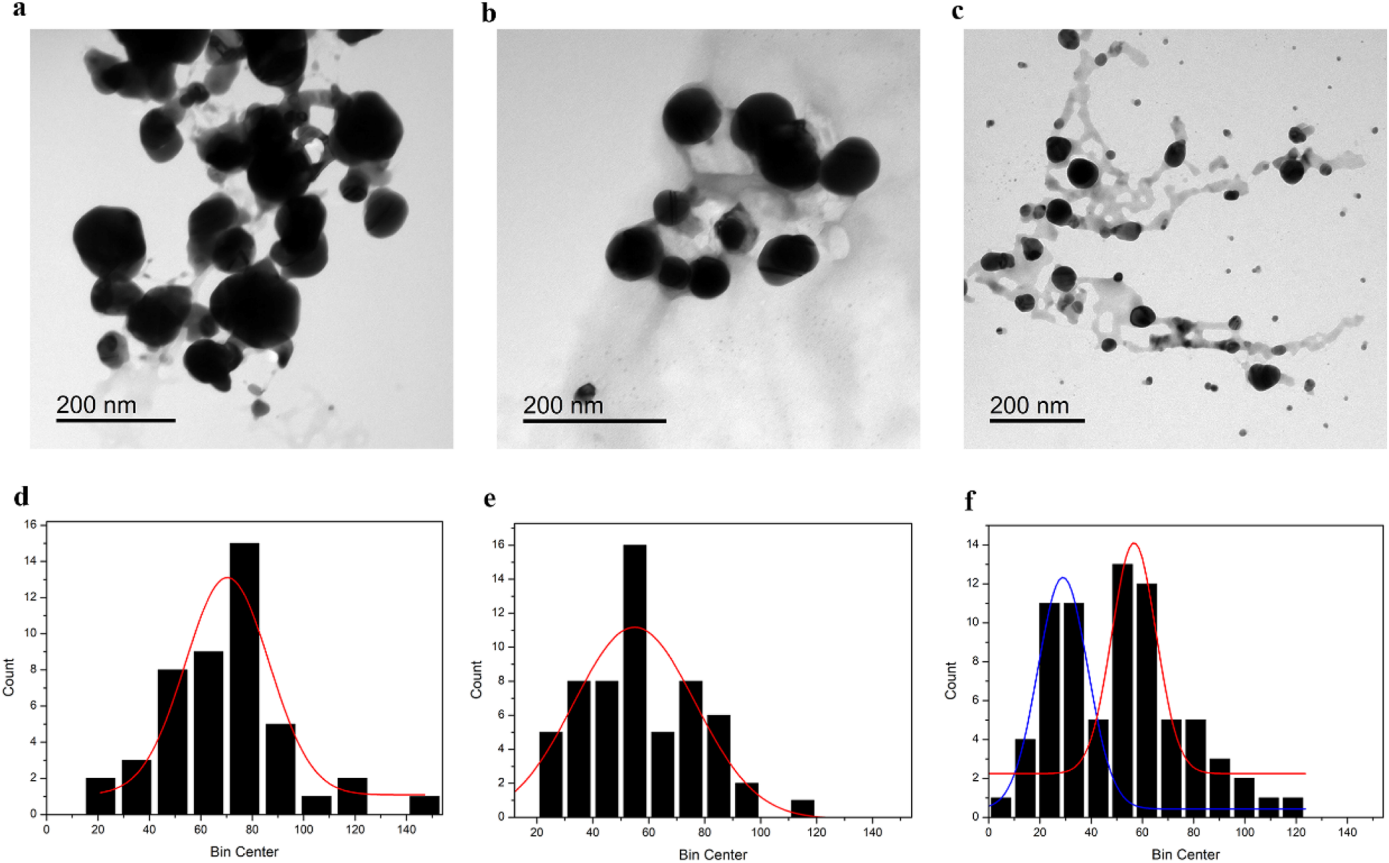
TEM acquisitions and corresponding particle size distributions of AgNPs synthesized in CS solutions through UV exposure for 15 min (a, d), 45 min (b, e), and 90 min (c, f).

All solutions, including CTRL, CS/UV, and CS0, were cast in polystyrene Petri dishes and dried at 35 °C for 48 h. Subsequently, they were stored in a controlled dark environment at (18 ± 2) °C and a relative humidity of (50 ± 5)% for 1 week. The UV-irradiated samples were placed in an isolated chamber, positioned 12.5 cm away from the lamp source, under room temperature and constant RH. Following the drying and cross-linking processes, the films were peeled off and stored for an additional week. The UV-treated samples exhibited a distinct reddish coloration, in stark contrast to the transparent CTRL, CS/UV, and CS0 films (Figure 1d).

To investigate the efficiency in Ag^+^ reduction and to correlate the observed colorimetric changes with AgNP formation, UV–vis spectroscopy analysis was carried out on the obtained free-standing films in the wavelength range of 248–800 nm (Figure 3). The analysis aimed to assess the effective reduction of Ag^+^ in AgNPs and to evaluate the impact of the UV exposure time. The absorption spectra revealed that the CTRL sample exhibited negligible absorption in the vis region (400–800 nm), consistent with its high transparency, while a pronounced absorption was observed in the UV region (248–400 nm). Notably, UV radiation applied to the CS solution prior to the drying process did not significantly affect the optical properties of the resulting CS film, as evidenced by the CS/UV spectrum. Similarly, the presence of AgNO_3_ had no appreciable effect, as confirmed from the CS0 absorption spectrum. In contrast, the appearance of the absorption peak in the 400–450 nm range was associated with the characteristic surface plasmon resonance (SPR) of AgNPs,^38^ confirming their formation. In addition, the intensity of this SPR band increased with longer UV exposure, indicating a time-dependent enhancement in Ag^+^ reduction and a consequent increase in the number of AgNPs formed.

**Figure 3:**
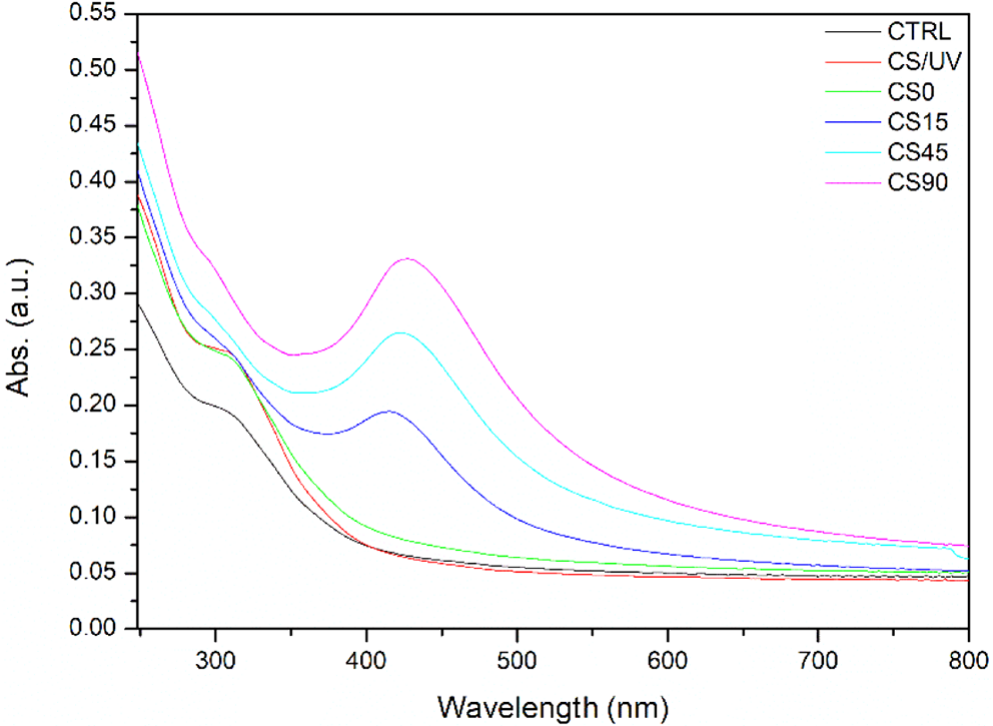
UV–vis analysis for chitosan film (CTRL), chitosan film exposed to UV lamps for 90 min (CS/UV), chitosan-Ag complex film (CS0), and chitosan-AgNP nanocomposites obtained through UV irradiation for 15, 45, and 90 min (CS15, CS45, CS90).

To further investigate how UV-induced AgNP formation affects surface properties, C.A. measurements were subsequently performed by the sessile drop method using Milli-Q as the probe liquid (Figure 4). Medium molecular weight CS is generally considered a hydrophilic polymer (C.A. < 90°), and the measured value (89 ± 3)° on the pristine CS film (CTRL) was in line with the scientific literature.^15,63^ Upon the addition of silver nitrate to the CS solution, the formation of the CS@Ag complex (CS0) slightly increased the C.A. to (93 ± 3)°, although the change was not statistically significant (*p* > 0.05). No appreciable difference was observed in the CS15 sample, probably due to a limited irradiation time. Exposure for 15 min appeared insufficient to induce notable surface modifications or generate a substantial number of NPs, as supported by other characterization data. Conversely, significant changes in wettability were observed in the CS45 and CS90 samples, with C.A. values of (96 ± 2)° and (98 ± 3)°, which corresponded to an increase of approximately 8% and 10% with respect to the CTRL sample. Interestingly, a comparable increase in C.A. was also recorded for the CS/UV sample after 90 min of UV exposure. This effect could be attributed to the photooxidation of the CS matrix under UV radiation, potentially leading to the formation of reactive species on the biopolymer chains. These active radicals, possibly generated prior to drying, may have promoted cross-linking between the polymer chains, thereby enhancing hydrophobicity.^46,64^ However, in the irradiated Ag-containing samples, the concurrent formation of AgNPs and their binding to reactive sites may have partially protected CS from UV-induced photooxidation. This protection might reduce the cross-linking rate and limit the increase in C.A. The effect could be attributed to the AgNPs’ surface plasmon absorption peak (λ ≈ 360 nm), as supported by the spectroscopic analysis.

**Figure 4:**
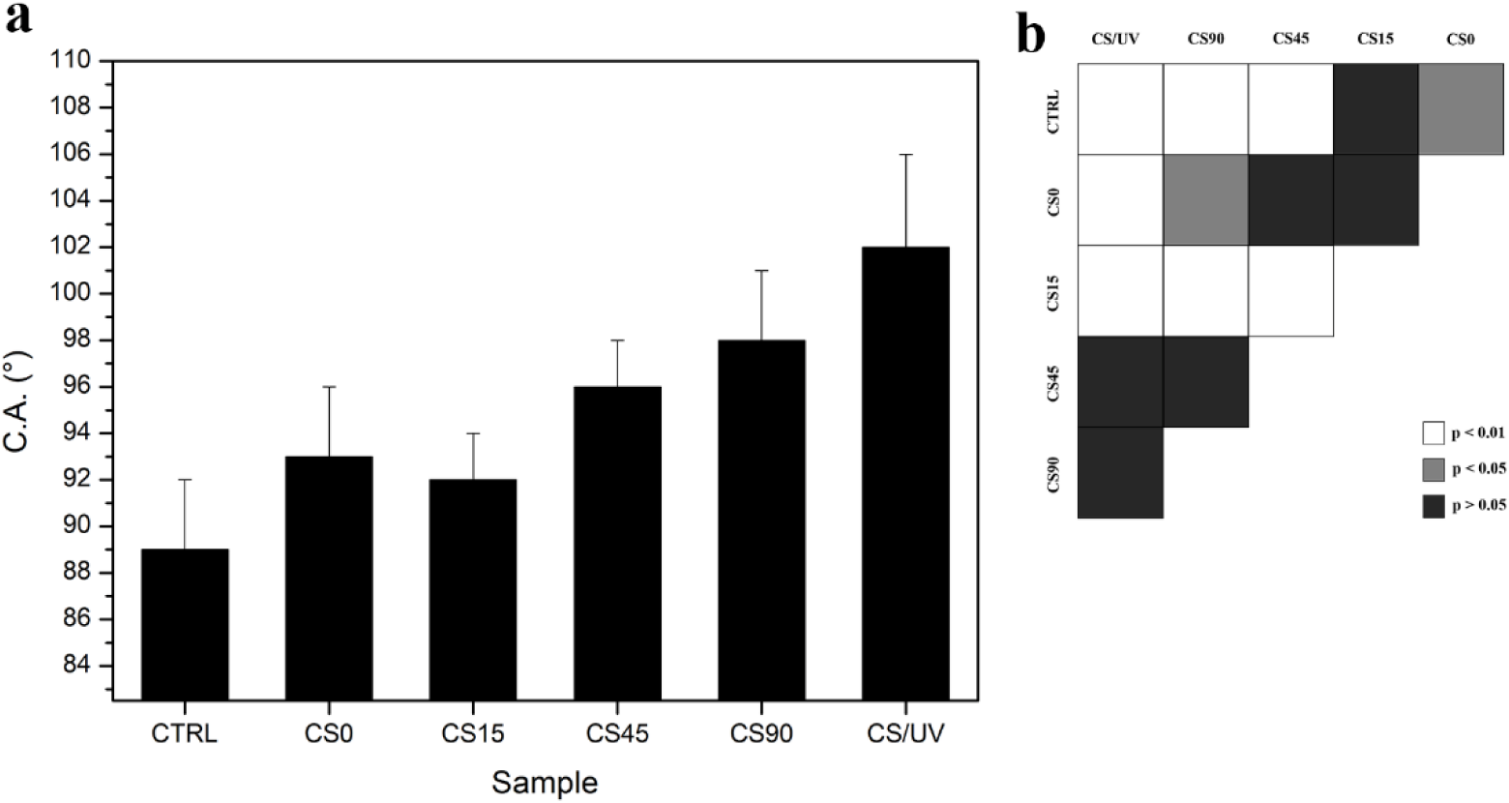
(a) Contact Angle (C.A.) measurements of the tested films performed by means of an optical tensiometer using the sessile drop technique; (b) statistical analysis for pairwise comparisons: white means *p* < 0.01, gray means *p* < 0.05, and black means *p* > 0.05, *n* = 60.

An alternative explanation involves the growth of AgNPs on the film surface, which may have affected the surface roughness. To investigate this hypothesis, surface roughness was quantified in terms of root mean square (R_q_) (Figure 5) through the analysis of topographic acquisitions obtained via AFM measurements (Figure 6).

**Figure 5:**
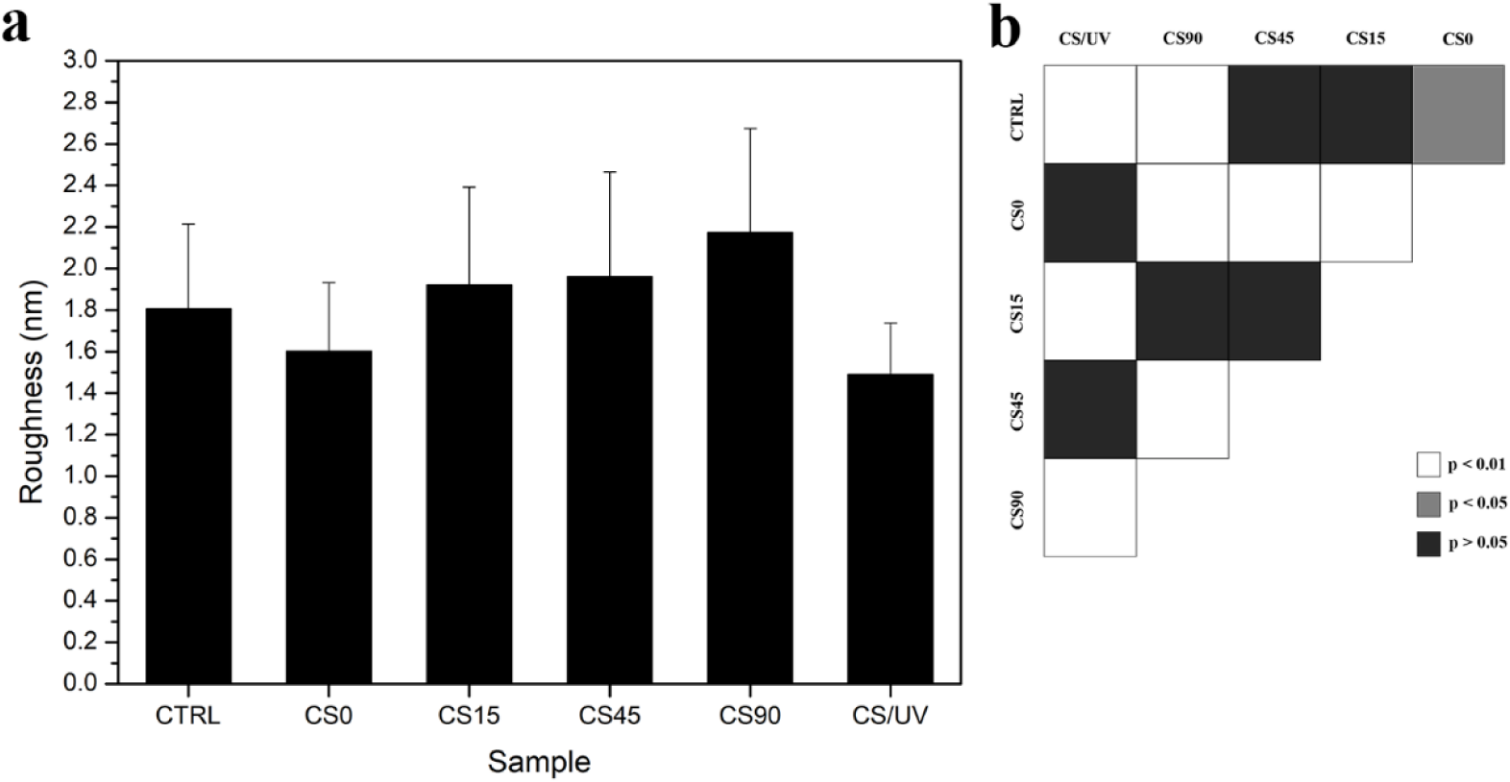
(a) Roughness values expressed as root-mean-square (R_q_) obtained by means of Atomic Force Microscopy (AFM) in Contact Mode for chitosan (CTRL), chitosan-silver complex (CS0), chitosan-AgNPs (CS15, CS45, CS90) for three UV exposure times (15, 45, 90 min), and chitosan exposed to UV for 90 min (CS/UV); (b) statistical analysis for pairwise comparisons: white means *p* < 0.01, gray means *p* < 0.05, and black means *p* > 0.05, *n* = 60.

**Figure 6:**
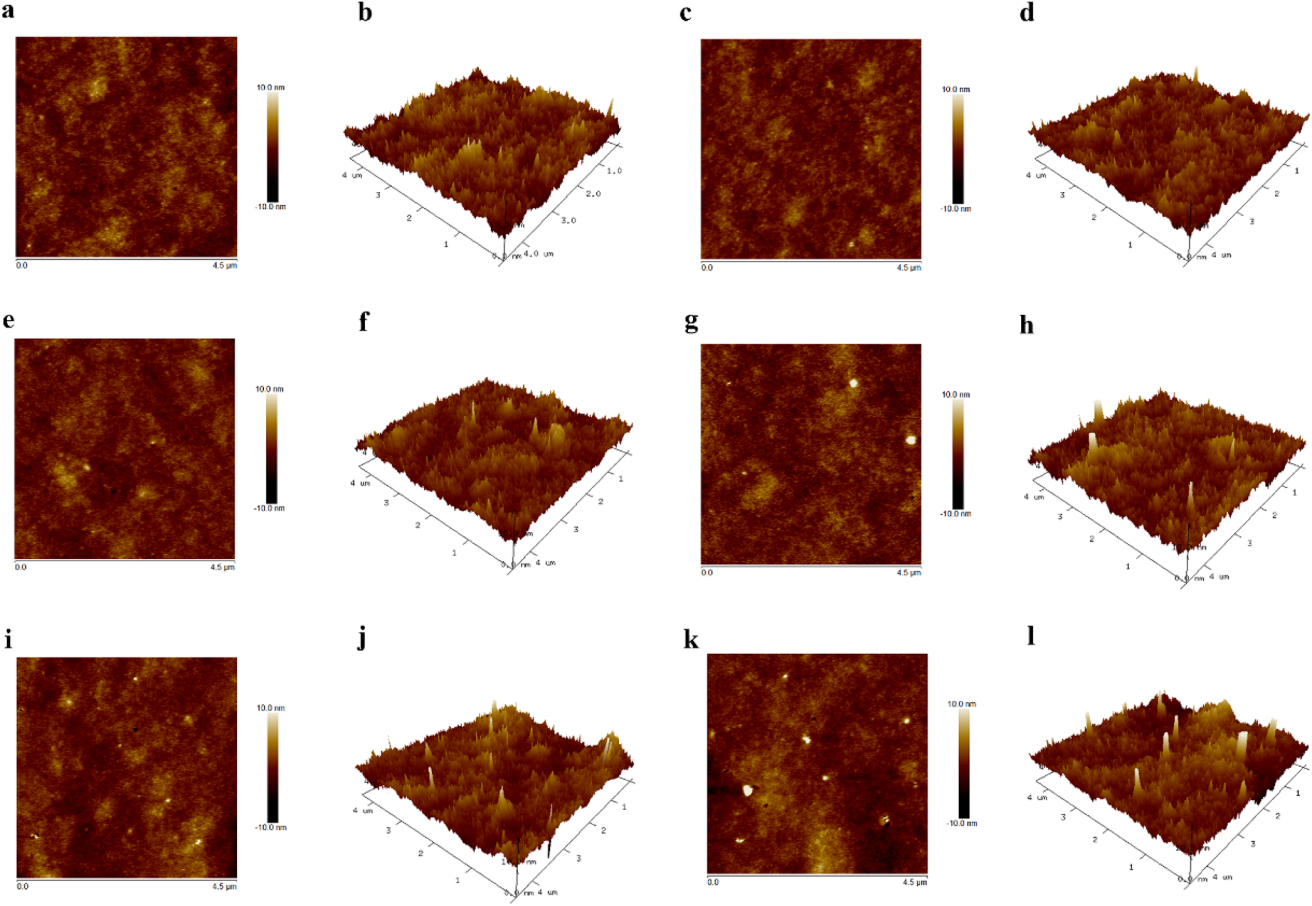
Representative 2D and 3D topographies of (a, b) chitosan films (CTRL), (c, d) chitosan UV-exposed for 90 min (CS/UV), (e, f) chitosan-silver complex (CS0), chitosan-AgNP *in situ* nanocomposites obtained after UV exposure for (g, h) 15 min, (i, j) 45 min, and (k, l) 90 min carried out by means of Atomic Force Microscopy (AFM), dimensions of 4.5 μm × 4.5 μm, height bar [−10, 10] nm.

For the pure CS film, the obtained values were consistent with those previously reported in the literature,^46,65^ including the slight decrease observed after UV exposure.^44^ The CS-Ag complex film CS0 exhibited statistically significantly lower R_q_ than CTRL (*p* < 0.05). The R_q_ increased proportionally with the UV exposure time and, consequently, with the number of formed AgNPs. As previously assumed, the increase in R_q_ could explain the difference in C.A. for CS90 and CS/UV samples, which were exposed to UV for the same duration. Indeed, the R_q_ obtained for the CS/UV sample was the lowest, probably due to polymer chain rearrangement following free radical formation. The reduced R_q_ for CS/UV compared with CS90 was attributed to the absence of NPs.

To complement the surface water resistance determined by the C.A. measurements, bulk water resistance properties, including moisture content (MC%) and the swelling degree (SD%), were evaluated, as they represent key factors for the suitability of materials in food packaging applications.^5^

Moisture uptake capacity, assessed through MC% measurements (Figure 7a), indicated that the addition of AgNO_3_ to the CS solution did not significantly affect the water absorption behavior of the film. CTRL and CS0 showed comparable values: (18.1 ± 2.9)% and (18.3 ± 3.3)%, respectively (*p* > 0.05). However, the formation and growth of AgNPs resulted in a slight decrease in MC%, as observed in CS15 (17.8 ± 2.6)%, CS45 (17.3 ± 2.3)%, and CS90 (17.4 ± 2.3)%. The lowest value was recorded for CS45, although this reduction was not statistically significant. In contrast, CS/UV films exhibited a statistically significant increase in MC%, with an average rise of ∼4% compared to both the CTRL and nanocomposite films.

**Figure 7:**
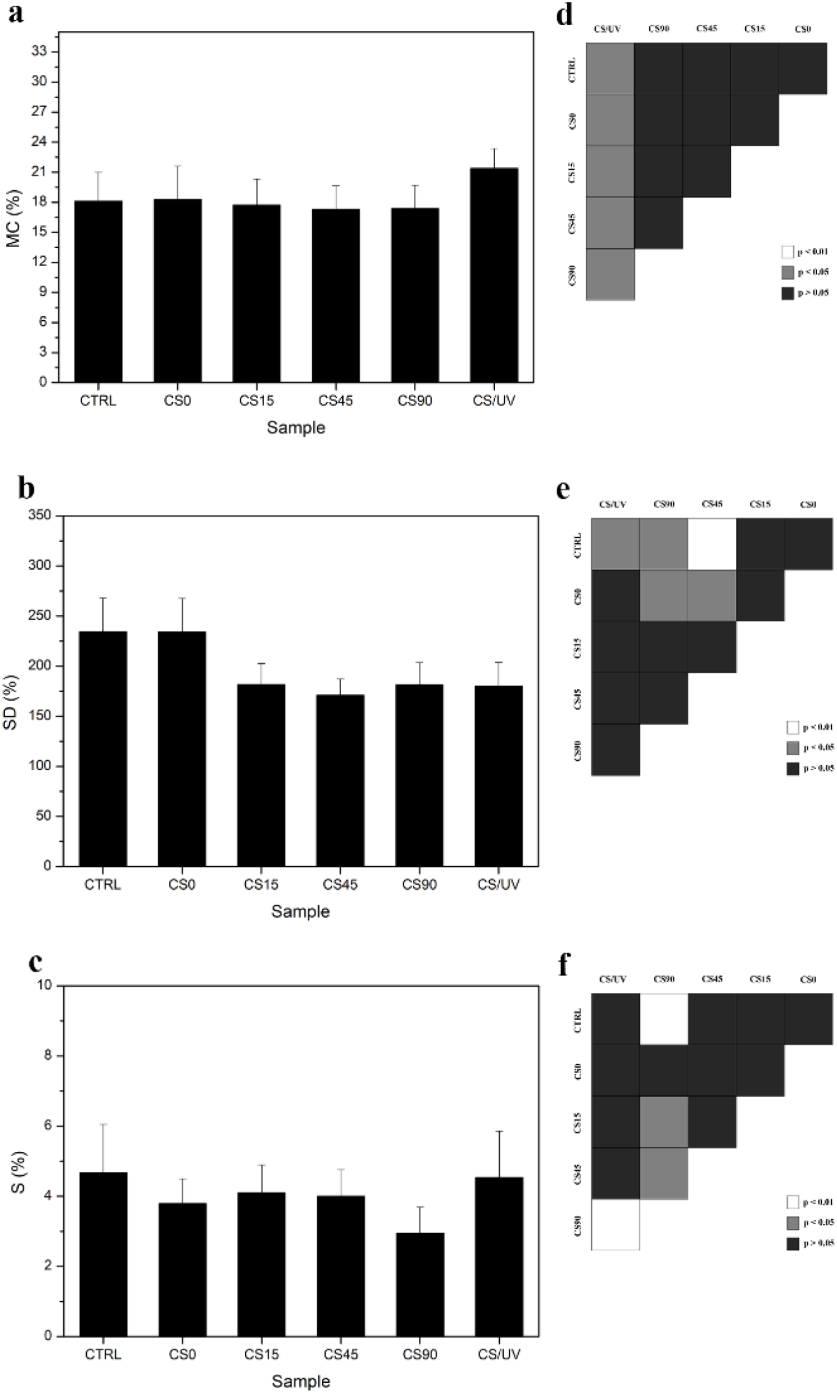
Mean values obtained for (a) Moisture Content (MC%), (b) Swelling Degree (SD%), and (c) Solubility (S%) for chitosan (CTRL), chitosan exposed to UV radiation (CS/UV), chitosan-silver complex (CS0), and nanocomposites chitosan-AgNPs obtained by UV photoinduced *in situ* reduction under exposure for 15, 45, and 90 min (CS15, CS45, and CS90); (d, e, f) statistical analysis for pairwise comparisons: white means *p* < 0.01, gray means *p* < 0.05, and black means *p* > 0.05, *n* = 8.

Differently, SD% measurements (Figure 7b) revealed that all samples containing AgNPs exhibited a significant reduction compared to the CTRL (235 ± 33%)%, with values ranging from 53% to 64%, in agreement with existing literature.^66,67^ Specifically, CS15, CS45, and CS90 showed SD% values of (182 ± 21%)%, (171 ± 16%)%, and (182 ± 22%)%, respectively. This reduction was consistent with the observed increase in surface water resistance measured via C.A. and was attributed to the ability of AgNPs to bind to the hydrophilic hydroxyl and amine groups of CS, limiting the polymer hydration.^37^ In contrast, the CS0 sample, which contained AgNO_3_ but no formed NPs, did not show any reduction in SD%, resulting in a value equal to (234 ± 33%)%. This finding, supported by previous C.A. results, suggested that Ag^+^ ions had minimal impact on the water resistance of CS. This could be attributable to the higher AgNO_3_ content in CS0, which exhibited hydrophilic behavior,^34^ whereas the conversion to AgNPs in nanocomposites resulted in a reduced amount of AgNO_3_ and a decrease in SD%, improving the water resistance of the resulting films.

It is known how UV irradiation also contributes to the reduction in SD% by promoting cross-linking between polymer chains.^37,67^ However, the SD% values recorded for CS90 and CS/UV were not significantly different. This suggests that the effects of AgNPs and UV-induced cross-linking may compete with the modification of the material structure.

The lowest SD% value was observed for CS45, which exhibited a 64% reduction compared to CTRL, representing the most pronounced improvement in water resistance among the nanocomposites.

This trend was further supported by S% analysis (Figure 7c), which provided complementary insight into the film’s water stability. In particular, the solubility value indicated that films cross-linked solely by UV were more susceptible to water-induced degradation than those containing AgNPs. Indeed, no statistically significant difference was found between CTRL (4.7 ± 1.4)% and CS/UV samples (4.5 ± 1.3)%, while lower S% values were observed for CS0 and all nanocomposite samples, specifically (3.6 ± 0.8)%, (4.1 ± 0.8)%, (4.0 ± 1.3)%, and (3.0 ± 0.7)% for CS0, CS15, CS45, and CS90, respectively. This result suggested that while the UV-induced cross-linking process could reduce the surface hydrophilicity, it was less effective in enhancing long-term resistance to water immersion compared to the presence of AgNPs. The lowest S% value recorded in CS90 was associated with the higher content of AgNPs, whose strong interaction with the polymer chains likely limited the exposure of hydrophilic groups and reduced their interaction with water molecules.^34^

To assess the impact of AgNP incorporation and UV treatment on the mechanical performance of the manufactured films, tensile tests were carried out, and results were reported in terms of tensile strength (TS), elongation at maximum force (ε_r_), and Young’s modulus (E) (Figure 8). The stress–strain curve of the CTRL sample was found to be characterized by a linear elastic zone, followed by a nonelastic region and then rupture, typical of air-dried chitosan films (Figure S1).^68,69^ The CTRL sample exhibited the lowest mechanical performance with TS = (43 ± 4) MPa, ε_r_ = (2.8 ± 0.2)%, and E = (1782 ± 102) MPa. A significant improvement in all of the investigated parameters was observed after the addition of AgNO_3_. In particular, CS0, CS15, CS45, and CS90 were found to be characterized by a linear elastic region, followed by a nonelastic region, strain hardening with necking, and rupture (Figure S1). As supported by literature data, the change in the mechanical behavior of the CS film could be clearly ascribable to the contribution of AgNO_3_ salt.^70^ CS0 films showed TS = (62 ± 11) MPa, ε_r_ = (4.6 ± 0.8)%, and E = (1921 ± 137) MPa. These enhancements were retained in the nanocomposite samples, with CS15, CS45, and CS90 showing comparable tensile strength: TS = (62 ± 5) MPa, TS = (62 ± 6) MPa, and TS = (61 ± 8) MPa, respectively (see S1). Similarly, the ε_r_ increased in the presence of NPs without significant difference with respect to different UV-time exposure, resulting in ε_r_ = (5.1 ± 0.8)% for CS0, ε_r_ = (4.7 ± 0.6)% for CS45, and ε_r_ = (5.7 ± 1.2)% for CS90. The slight increase in ε_r_ observed for CS90 suggested that the larger amount of NPs conferred to the polymeric matrix of the films more resistance to elongation. The most pronounced mechanical improvement was obtained for the CS/UV sample, which reached TS and ε_r_ values of (74 ± 8) MPa and (6.7 ± 1.0)%, respectively. Here, an extended strain hardening phase was observed, attributable to the stretching of cross-linked chitosan chains.

**Figure 8:**
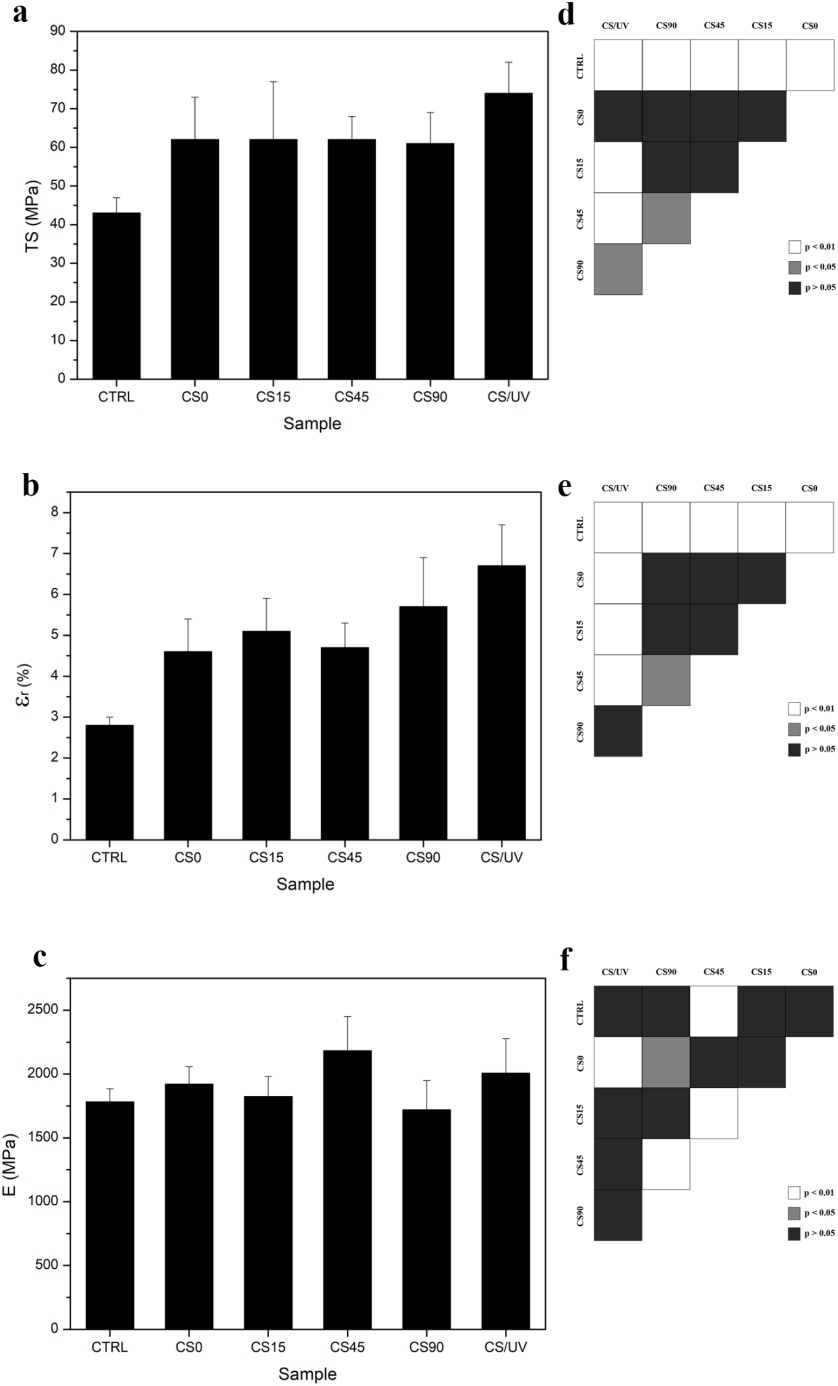
Reported values for (a) Tensile Strength (TS), (b) Elongation at maximum TS (ε_r_) and (c) Young’s modulus (E) for the manufactured chitosan films (CTRL), chitosan UV-exposed films (CS/UV), chitosan-silver complex films (CS0), and chitosan-AgNP nanocomposite films (CS15, CS45, and CS90); (d, e, f) statistical analysis for pairwise comparisons: white means *p* < 0.01, gray means *p* < 0.05, and black means *p* > 0.05, *n* = 8.

Furthermore, the E analysis demonstrated how this parameter becomes slightly higher with respect to the CTRL samples, remaining approximately the same among all the treated samples: (1921 ± 137) MPa, (1824 ± 158) MPa, (2182 ± 269) MPa, and (1720 ± 230) MPa for CS0, CS15, CS45, and CS90 (*p* > 0.05), respectively (Figure 8c).

The sensitivity of CS to UV irradiation has previously been reported, particularly in relation to its mechanical properties. Prolonged exposure of CS films to UV light has been shown to induce photodegradation, resulting in a reduction of TS and elongation properties. However, such findings were limited to CS films polymerized prior to UV exposure and irradiated at wavelengths within the damage range (λ < 300 nm).^44,45,71^ Consistent with the literature, our study also confirmed the sensitivity of CS to UV radiation. Nevertheless, under the specific manufacturing conditions employed in this work, UV treatment led to improvements in mechanical performance. As discussed previously, this effect could be attributable to the formation of cross-links generated either by UV radiation or by the presence of AgNPs.

The antibacterial activity of the 6 mm-diameter films was evaluated against *E. coli* (Gram-negative) and *S. aureus* (Gram-positive) through the agar diffusion method (Figure 9 b,c,d,e), as two representative bacteria commonly encountered in the agrifood fields.^72^ After 24 h, pure CS film, both untreated and irradiated, exhibited no antibacterial activity (Figure 9a); this is in agreement with previous reports documenting low or negligible efficacy of CS-based films against *E. coli* and *S. aureus* under similar conditions.^55,73^

**Figure 9:**
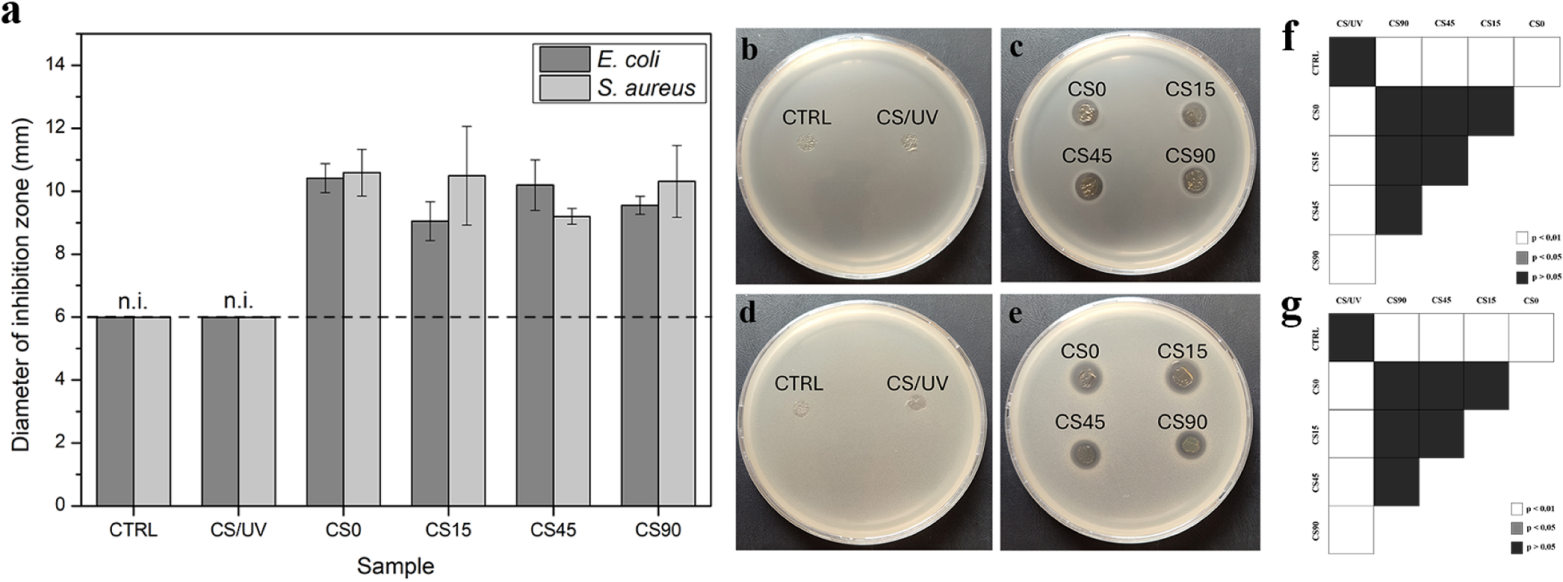
(a) Antibacterial activity measured on *n* = 3 tests and values obtained as Mean ± ε, where ε is the maximum error. Representative images of inhibition disks for (b,c) *E. coli* and (d,e) *S. aureus* ; (b,d) chitosan (CTRL) and chitosan-UV-exposed films (CS/UV); (c,e) chitosan-silver complex (CS0) and chitosan-AgNP *in situ-*obtained nanocomposites (CS15, CS45, and CS90). Six millimeters is the diameter of the disk (dotted line) and indicates no inhibition (n.i.) zone around the disk (CTRL, CS/UV); (f, g) statistical analysis for pairwise comparisons respectively for the diameter of the inhibition zone for *E. coli* and *S. aureus*: white means *p* < 0.01, gray means *p* < 0.05, and black means *p* > 0.05, *n* = 3.

Conversely, all films containing AgNO_3_ displayed measurable antibacterial activity against both bacteria. Notably, no significant correlation was observed between the UV exposure time and inhibition efficacy. All Ag-containing samples, including the chitosan-silver complex (CS0) and nanocomposites (CS15, CS45, and CS90), showed a similar rate of inhibition (Figure 9a).

It is well-established that the antibacterial efficacy of Ag is primarily associated with the release of Ag^+^ ions rather than the presence of AgNPs.^74^ This explains the inhibition halos observed even in CS0 films, which contain Ag^+^ ions but not NPs.

Upon contact with bacterial cells, Ag^+^ ions released from CS@Ag films disrupt the bacterial cell wall and membrane integrity, ultimately leading to irreversible cellular damage.^30^ Although the antibacterial activity of biomaterials for food packaging applications is often preliminarily evaluated by the agar diffusion method,^51,75^ some limitations have to be taken into account, such as the complexity of real food matrices, the storage conditions, and the kinetic release influenced by the chemical composition of food, which can affect the release of the antimicrobial agent.^76^

AgNPs in food coatings are widely used for their well-studied antimicrobial properties, which help extend the shelf life and enhance the safety of food matrices. Composite films with the addition of AgNPs as food preservatives have been previously tested with various food matrices, including fruits, vegetables,^77,78^ and meat.^79,80^ While AgNPs offer a broad-spectrum antimicrobial action useful for food preservation, optimizing NP release and ensuring compliance with safety regulations is essential for applications with food matrices.^81^ As recently discussed by Jangid et al.,^82^ various regulatory agencies have provided guidelines for the use of nanomaterials in food packaging, such as the Commission Regulation (EU) No 10/2011, which requires toxicological assessment and evaluation of the safety profile as essential tools to promote the use of AgNPs as safe antibacterial agents. For a complete evaluation of the efficacy of the developed CS-based films as smart materials for food preservation, further experiments will be conducted to assess the antibacterial action against common foodborne pathogens, like *Pseudomonas aeruginosa*,^83^ *Salmonella* spp.,^84^ and *Listeria monocytogenes*.^85^

However, when AgNPs are formed within the polymeric matrix, the release of Ag^+^ ions is expected to be slower and more sustained, potentially offering longer-lasting antibacterial activity compared to films containing only silver salt.^38,74^ To investigate this hypothesis, circular film samples were fully immersed in 1 mL of Milli-Q water (pH 7) at room temperature for 15 days. The concentration of Ag^+^ ions released into the solution was quantified by ICP-OES measurement after 1, 7, and 21 days (Figure 10). At each time point, the solution was collected and digested overnight with 0.5 mL of HNO_3_. The samples were reimmersed in freshly Milli-Q water, and the same procedure was repeated for the next intervals. After 24 h, the amount of Ag^+^ ions released (Figure 10) was negligible for all the samples investigated. Instead, for longer immersion times, a higher release of Ag^+^ was recorded for CS0 samples compared to all AgNPs-containing nanocomposites after both 7 and 21 days. This behavior could be attributed to the higher mobility of ionic Ag, which is more readily desorbed from the polymeric matrix due to water penetration with reactive sites.^73^ Among CS@Ag samples, CS15 and CS45 exhibited similar Ag^+^ release profiles over the experiment time. Interestingly, CS90 showed a marked increase in Ag^+^ concentration after 15 days, surpassing that of both CS15 and CS45. This could be linked to the presence of smaller AgNPs, as suggested by TEM analysis (Figure 2), which are known to release Ag^+^ more efficiently than larger NPs due to their higher surface area-to-volume ratio.^86^ Furthermore, cross-linking effects induced by UV exposure and AgNPs may have contributed to slowing the ion diffusion rate in the liquid medium.^87^

**Figure 10:**
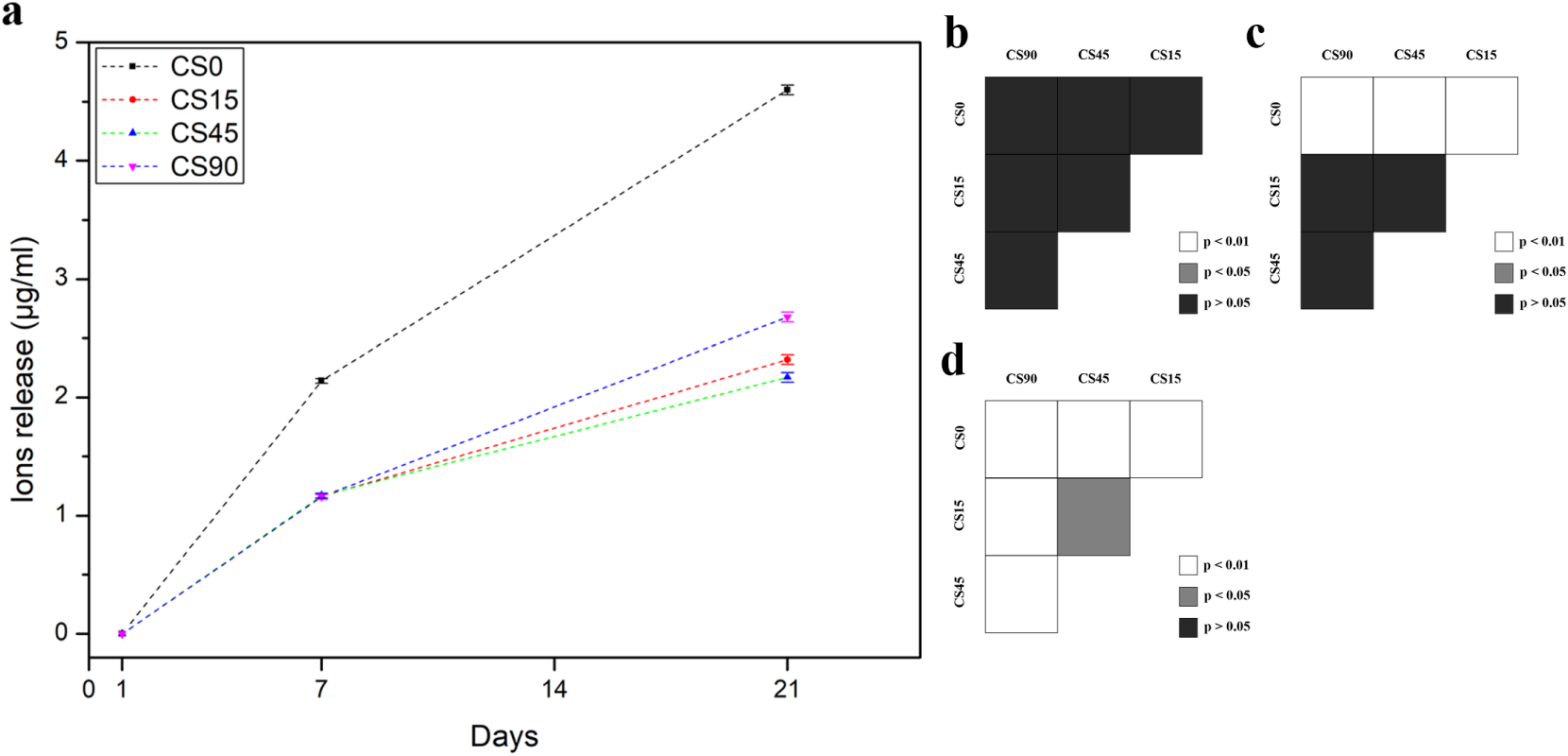
(a) Silver ions (Ag^+^) release in Milli-Q water (pH 7) at room temperature for the CS@Ag samples quantified by means of ICP-OES after 1 day, 7 days, and 21 days; (b, c, d) statistical analysis for pairwise comparisons respectively for ion release after 1 day, 7 days, and 21 days: white means *p* < 0.01, gray means *p* < 0.05, and black means *p* > 0.05, *n* = 3.

## 4 Conclusions

In the present work, CS@AgNP nanocomposite films were successfully fabricated through a simple and scalable one-step UV-assisted photoreduction method, in which CS simultaneously serves as both a reducing and capping agent and a polymeric matrix. The *in situ* formation of AgNPs was controlled by modulating the UV exposure time (15, 45, and 90 min), enabling the production of nanocomposites with tailored and tunable Ag content. This study demonstrates the effectiveness of the proposed method and the enhancement of physicochemical, mechanical, and antibacterial performances of CS@AgNP films compared to conventional CS-based materials reported to date.

UV irradiation played a dual role, simultaneously inducing NP formation and promoting cross-linking within the polymer network, allowing precise control over film properties without requiring additional chemical agents. These findings underscore the potential of CS@AgNP nanocomposites as a sustainable and efficient platform for active packaging applications, particularly in the agrifood field, where antimicrobial activity and water resistance are essential for ensuring food safety and extending shelf life. Overall, the proposed methodology provides a versatile and environmentally friendly route for designing high-performance bioactive films with potential applications extending beyond food packaging into broader areas of biomedical and environmental interest.

## Supplementary Material



## Notes

Tables with all the results obtained from the experiments described herein, together with their statistical analysis; a figure showing the original stress–strain curves (PDF)
