# Supplementary material for: Structural and Physical Properties of Chitosan Films Containing UV-Driven In Situ Growth of Silver Nanoparticles
Source: ACS Omega. 2026 Mar 30;11(13):20213–26. doi: 10.1021/acsomega.5c09873 (PMC13063177; doi:10.1021/acsomega.5c09873)
Supplement: Supplementary file 1 [file ao5c09873_si_001.pdf]

Structural and physical properties of Chitosan films containing UV-driven in-situ growth of Silver Nanoparticles

Daniele Costa[a], Mariafrancesca Cascione[a,b,*], Valeria De Matteis[c,**], Riccardo Di Corato[b,d], Nunzia Gallo[e], Stefania Villani[e], Christian Demitri[c], Pietro Alifano[c], Alessandro Sannino[c], Gianaurelio Cuniberti[f], Rosaria Rinaldi[a,b]

[a] *Department of Mathematics and Physics "Ennio De Giorgi", University of Salento, Via Arnesano, Lecce, 73100, Italy.*

[b] *Institute for Microelectronics and Microsystems (IMM), CNR, Via Monteroni, Lecce, 73100, Italy.*

[c] *Department of Experimental Medicine, University of Salento, Via Barsanti, Arnesano (LE), 73100, Italy.*

[d] *Center of Biomolecular Nanotechnologies, Istituto Italiano di Tecnologia, Via Monteroni, Lecce, 73100, Italy.*

[e] *Department of Engineering for Innovation, University of Salento, Via Monteroni, Lecce, 73100, Italy.*

[f] *Institute for Materials Science and Max Bergmann Center for Biomaterials TUD Dresden University of Technology, Dresden, Germany.*

Corresponding Authors*: mariafrancesca.cascione@unisalento.it; valeria.dematteis@unisalento.it

Statistical analysis

All the results in the present Supplementary file (S1) were reported as Means ± Standard Deviation. Statistical analyses were performed using OriginPro software (OriginLab, version 8.5, Northampton, MA, USA). Specifically, parametric (ANOVA and t-test) and non-parametric (Kruskal-Wallis H and Mann-Whitney) tests were used for groups or pairwise comparisons. Differences were considered statistically significant at $p < 0.05$. In detail, NPs size in Table S1, Contact Angle (C.A.), Moisture Content (MC%), Swelling Degree (SD%) and Solubility (S%) results were reported in Table S2; roughness measurements, expressed as root-mean-square roughness (R_q), were reported in Table S3. Tensile Strength (TS), Elongation at maximum TS (ε_r) and Young's Modulus (E) values were presented in Table S4 and the stress-strain curves reported in Figure S1. Finally, Table S5 contains the antibacterial activity expressed as Diameter of Inhibition zone, evaluated by agar diffusion method. Specifically, the same superscript letter was assigned to pairs of statistically equivalent results ($p > 0.05$); different superscript letters in the same column indicate a statistically significant difference between the involved groups ($p < 0.05$).

Sample name	NPs size (nm)
**CS15**	68 ± 22[a]
**CS45**	58 ± 21[b]
**CS90 (bottom plot)**	29 ± 10[c]
**CS90 (top plot)**	57 ± 9[b]

Table S1: Values for AgNPs size obtained after exposing the CS@AgNO3 solution for respectively 15, 45 and 90 minutes. Values are presented as Means ± Standard deviation (SD). n=60.

Sample name	C.A. (°)	MC%	SD%	S%
**CTRL**	89 ± 3^{a}	18.1 ± 2.9^{a}	235 ± 33^{a}	4.7 ± 1.4^{a}
**CS0**	93 ± 3^{b}	18.3 ± 3.3^{a}	234 ± 33^{ab}	3.6 ± 0.8^{a}
**CS15**	92 ± 2^{ab}	17.8 ± 2.6^{a}	182 ± 21^{abc}	4.1 ± 0.8^{a}
**CS45**	96 ± 2^{bc}	17.3 ± 2.3^{a}	171 ± 16^{cd}	4.0 ± 0.7^{a}
**CS90**	98 ± 3^{cd}	17.4 ± 2.3^{a}	182 ± 22^{cde}	3.0 ± 0.7^{b}
**CS/UV**	102 ± 4^{cd}	21.4 ± 2.0^{b}	180 ± 14^{bcde}	4.5 ± 1.3^{a}

Table S2: Values for Contact Angle (C.A.), Moister Content (MC%), Swelling Degree (SD%) and Solubility (S%) and obtained for the manufactured films: chitosan (CTRL), chitosan UV exposed (CS/UV), chitosan-silver complex (CS0), chitosan-AgNPs (CS15, CS45 and CS90). Values are presented as Means ± Standard deviation (SD). n=8 for C.A. and n=8 for MC%, SD% and S%.

Sample name	Rq (nm)
**CTRL**	1.8 ± 0.4^{a}
**CS0**	1.6 ± 0.3^{b}
**CS15**	1.9 ± 0.5^{ac}
**CS45**	2.0 ± 0.5^{acd}
**CS90**	2.2 ± 0.5^{cd}
**CS/UV**	1.5 ± 0.3^{b}

Table S3: Roughness values expressed as root-mean-square (R_q) obtained by means of Atomic Force Microscopy (AFM) in Contact Mode for chitosan (CTRL), chitosan-silver complex (CS0), chitosan-AgNPs (CS15, CS45, CS90) for three UV exposure time (15, 45, 90 min), and chitosan exposed to UV for 90 min (CS/UV). n=30.

Sample name	TS (MPa)	ε_r (%)	E (MPa)
**CTRL**	89 ± 3^{a}	18.1 ± 2.9^{a}	235 ± 33^{a}
**CS0**	93 ± 3^{b}	18.3 ± 3.3^{b}	234 ± 33^{ab}
**CS15**	92 ± 2^{bc}	17.8 ± 2.6^{bc}	182 ± 21^{abc}
**CS45**	96 ± 2^{bcd}	17.3 ± 2.3^{bcd}	171 ± 16^{bd}
**CS90**	98 ± 3^{bcd}	17.4 ± 2.3^{bcde}	182 ± 22^{ace}
**CS/UV**	102 ± 4^{b}	21.4 ± 2.0^{e}	180 ± 14^{abcde}

Table S4: Tensile Strength (TS), Elongation at maximum TS (ε_r) and Young's Modulus (E) for the manufactured samples. n=6.

Diameter of Inhibition zone (mm)		
**Sample name**	***E. coli***	***S. aureus***
**CTRL**	6 ± 0[a]	6 ± 0[a]
**CS0**	10.4 ± 0.5[b]	10.6 ± 0.7[b]
**CS15**	9.1 ± 0.6[b]	10.5 ± 1.6[b]
**CS45**	10.2 ± 0.8[b]	9.2 ± 0.3[b]
**CS90**	9.6 ± 0.3[b]	10.31 ± 1.1[b]
**CS/UV**	6 ± 0[a]	6 ± 0[a]

Table S5: Antibacterial activity measured by means of inhibition disk method for *E. coli* and *S.aureus*. n=3.

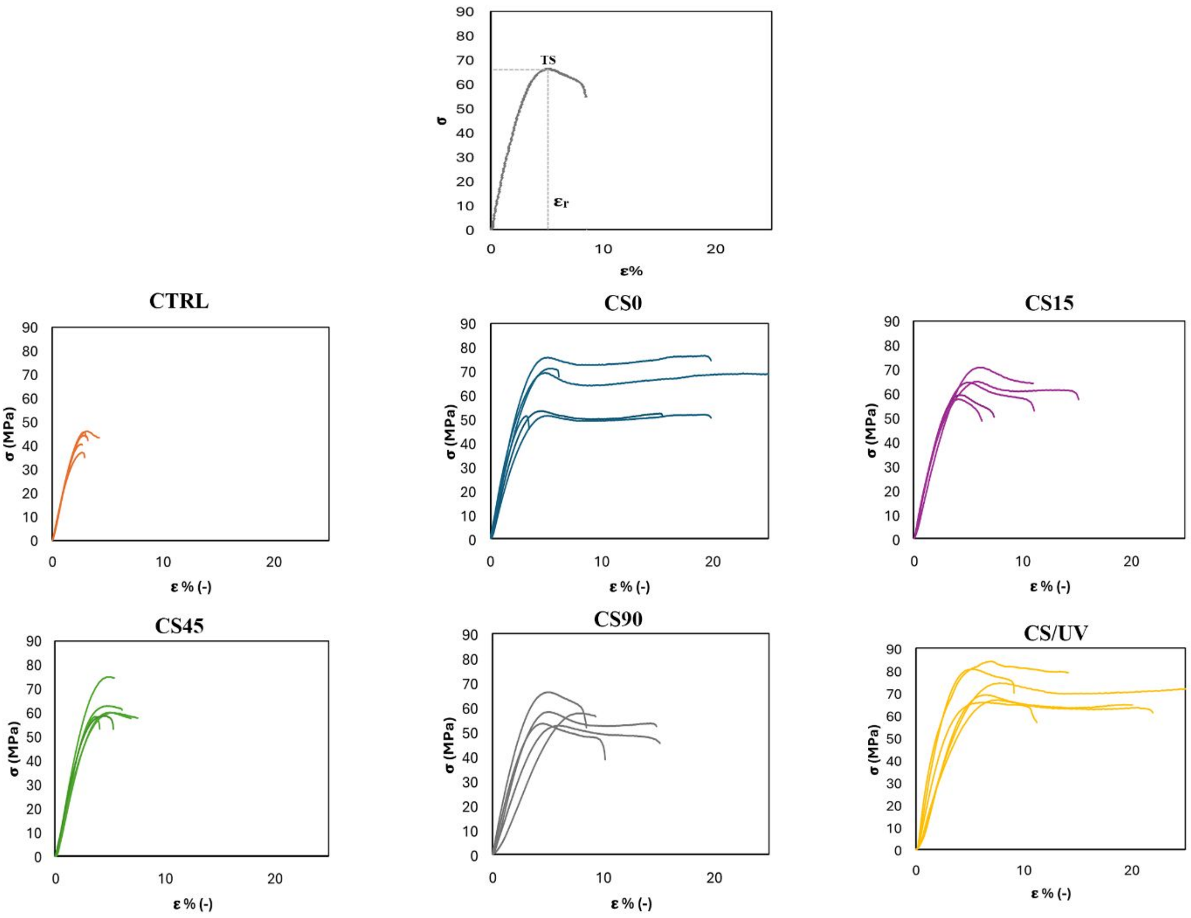

Figure S1: Stress/strain curves (σ/ε%) obtained in accordance with ASTM D882 standard practice, using a ZwickLine universal testing machine (Zwick/Roell, Ulm, Germany).
